# Geospatial Health (GeoHealth): Current Trends, Methods, and Applications

**DOI:** 10.3390/tropicalmed8070366

**Published:** 2023-07-17

**Authors:** Frank Badu Osei, Santanu Sasidharan

**Affiliations:** 1 Faculty of Geo-Information Science and Earth Observation (ITC), University of Twente, 7500 AE Enschede, The Netherlands; 2 Department of Physics and Astronomy, University of British Columbia, Vancouver, BC 2329, Canada; santanu.sasidharan@ubc.ca

As an emerging field, Geospatial Health (GeoHealth) integrates geospatial technologies, (spatial) epidemiology, and health services/resource allocations (health accessibility), with a focus to fight the burden of diseases. Many diseases have become a global burden. According to the global burden of diseases (GBD) in 2019, an estimated six infectious diseases were among the top ten causes of disability-adjusted life years (DALYs) in children younger than 10 years. These include lower respiratory infections, diarrheal diseases, malaria, meningitis, whooping cough, and sexually transmitted infections.

While some diseases may only ravage local populations, their economic burdens have global impacts. For instance, underdeveloped and developing countries rely on advanced countries to support their health resources to sustain their annual budgets. Additionally, vertical transmission via global trade and economic routes can lead to local diseases turning into global pandemics; the recent COVID-19 pandemic is an example. Therefore, local actions, based on reliable assessment and population health status information, need to be strengthened by improving the surveillance and assessment of trends and risks, development of applicable intervention methods, and optimization and allocation of health resources. Local-level monitoring and evaluation leading to local actions is, therefore, the key to reducing the global burden of diseases. Hence, this Special Issue focuses on the current trends and advances in the development, application, and integration of geospatial technologies, disease epidemiology, and demography to combat infectious diseases.

In this Special Issue, there are three dedicated articles on the application of geospatial health to SARS-CoV-2. Tzeng (2023) discusses the effectiveness of lopinavir/ritonavir in the treatment of SARS-CoV-2. The author demonstrated an approach to refine the binary outcomes of the two drugs according to geographical diversity [1]. The second study by Saita et al. involves the temporal and spatial cluster variations of dengue in Thailand. With the help of a seasonal autoregressive integrated moving average (ARIMA) model, the study demonstrated that dengue cases sharply decreased during the COVID-19 pandemic, and spatial clusters were lower when compared to the pre-pandemic environments in Thailand [2]. Furthermore, an insightful COVID-19 case report illustrates the use of community engagement data and geographic information systems to enhance vaccination efforts in ethnic minority groups in the United States [3]. Zhou et al. critically analyzed the geospatial distribution of health resources in China, highlighting a mismatch in resource allocation. The article also suggests the need for policy reframing to justify spatial adaptation and spatial equity, and optimize the allocation of health resources [4].

The Special Issue further focuses on the implementation of GeoHealth in viral diseases. Jagadesh et al. employed a biogeographic approach to determine the factors and predict future hotspots for three virus families: the *Filoviridae*, *Coronaviridae*, and *Henipavirus* genera. The study indicated that the common factors influencing disease emergence were climatic variables and human-induced land modifications [5]. Another study assessed the risk of dengue cases in Indonesia in relation to multi-factorial climate change. The study elucidated that an increased risk of dengue was observed in the years affected by El Nino and La Nina climate extremes, additionally predicting the regions that are vulnerable to dengue risks influenced by climate [6]. A systematic review of the Oropouche virus, an emerging vector-borne arbovirus, was documented for its presence in Latin America by Sciancalepore et al., whereby the One Health approach and geospatial techniques were combined [7]. The review suggested the presence of the virus in humans, four vector species, four genera of non-human primates, and one species of sloth.

In addition to viral infections, this Special Issue includes studies on other infectious diseases such as leishmaniasis, tuberculosis, trypanosomiasis, cholera, helminths, and scabies. Maule et al. analyzed the spatiotemporal pattern and climatic determinants of visceral leishmaniasis in Italy [8]. The findings of the study indicated the endemicity of visceral leishmaniasis in the Italian peninsula, and that climatic factors influence the geographical distribution of the cases. Another study on trypanosomiasis, a related parasitic protozoan, showed that targeted policy interventions are required in Zambia’s healthcare systems to improve the detection and management of infectious diseases [9]. Among the two studies on tuberculosis in this Special Issue, Yu et al. analyzed the diagnostic performance of QFG-GIT and the need for personalized cutoff values in diagnosis [10], while Scholze et al. reported the hotspot regions with tuberculosis and alcohol, tobacco, and other drugs in southern Brazil using a geospatial intelligence application. The evidence suggested the existence of an association between tuberculosis and drug addiction within the investigated hotspot regions [11]. The use of geospatial videos to map environments with cholera risk in Congo was conducted by Curtis et al., highlighting the use of data science and machine learning to detect infectious diseases in health-risk regions [12]. Nyandwi et al. also detected the spatial distribution of parasitic helminth diseases in Rwanda and associated the parasite incidence with soil characteristics, rainfall, wetlands, population density, and proportion of the rural disease burden [13]. Another interesting article in this Special Issue is the opinion piece by Glennie et al., discussing the importance of a community-led model for scabies surveillance in remote Aboriginal communities in Australia [14].

This Special Issue encompasses a variety of articles on the use of geospatial health to fight infectious diseases and formulate policy decisions. The findings in the studies strongly suggest that the consideration of human-induced environmental changes can cause significant shifts in the geographical distribution of infectious diseases. Further research on these topics is needed to advance the field and prevent future endemic and epidemic outbreaks. We take this opportunity to thank the excellent authors, critical reviewers, and editorial staff who contributed to this Special Issue.
